# Persistent flocks of diverse motile bacteria in long-term incubations of electron-conducting cable bacteria, *Candidatus* Electronema aureum

**DOI:** 10.3389/fmicb.2023.1008293

**Published:** 2023-02-23

**Authors:** Jamie J. M. Lustermans, Jesper J. Bjerg, Laurine D. W. Burdorf, Lars Peter Nielsen, Andreas Schramm, Ian P. G. Marshall

**Affiliations:** ^1^Section for Microbiology, Department of Biology, Center for Electromicrobiology, Aarhus University, Aarhus, Denmark; ^2^Department of Biology, Microbial Systems Technology Excellence Centre, University of Antwerp, Wilrijk, Belgium

**Keywords:** flocking, cable bacteria, electron conduction, succession, interspecies electron transfer, time series, microprofiles

## Abstract

Cable bacteria are centimeters-long filamentous bacteria that oxidize sulfide in anoxic sediment layers and reduce oxygen at the oxic-anoxic interface, connecting these reactions *via* electron transport. The ubiquitous cable bacteria have a major impact on sediment geochemistry and microbial communities. This includes diverse bacteria swimming around cable bacteria as dense flocks in the anoxic zone, where the cable bacteria act as chemotactic attractant. We hypothesized that flocking only appears when cable bacteria are highly abundant and active. We set out to discern the timing and drivers of flocking over 81 days in an enrichment culture of the freshwater cable bacterium *Candidatus* Electronema aureum GS by measuring sediment microprofiles of pH, oxygen, and electric potential as a proxy of cable bacteria activity. Cable bacterial relative abundance was quantified by 16S rRNA amplicon sequencing, and microscopy observations to determine presence of flocking. Flocking was always observed at some cable bacteria, irrespective of overall cable bacteria rRNA abundance, activity, or sediment pH. Diverse cell morphologies of flockers were observed, suggesting that flocking is not restricted to a specific, single bacterial associate. This, coupled with their consistent presence supports a common mechanism of interaction, likely interspecies electron transfer *via* electron shuttles. Flocking appears exclusively linked to the electron conducting activity of the individual cable bacteria.

## 1. Introduction

Cable bacteria are multi-cellular filaments that can become several centimeters in length and can be found globally in freshwater and marine sediments (Pfeffer et al., 2012; Risgaard-Petersen et al., 2015; Burdorf et al., 2017; Liu et al., 2021; Xu et al., 2021). Electrons are transported upwards through the filament and this results in their ability to physically separate the oxidation of sulfide in the deeper layers from oxygen respiration at the oxic zone. Cable bacteria have a strong influence on sediment geochemistry including the formation of a gradually widening suboxic zone between the retreating oxygen penetration depth (OPD) and increasing sulfide appearance depth (SAD; Pfeffer et al., 2012; Risgaard-Petersen et al., 2012; Meysman et al., 2015; Liu et al., 2021). This is coupled with a distinctive pH peak, congruent with the cable bacteria’s activity and their growth pattern, which is described by a lag phase that rapidly changes into an intense exponential phase and it ends in a trailing decline (Pfeffer et al., 2012; Schauer et al., 2014; Xu et al., 2021).

Besides geochemistry, the activity of cable bacteria has been shown to also influence associated microbial communities, such as iron-cycling bacteria and sulfur-oxidizing Epsilon-and Gammaproteobacteria in marine sediments (Vasquez-Cardenas et al., 2015; Otte et al., 2018). The sulfide oxidizers were found to be active below the oxic zone and were proposed to connect with the cable bacteria’s electron transport through the oxidation of sulfide and subsequent transfer of the gained electrons to cable bacteria (Vasquez-Cardenas et al., 2015). Another example of association are flocking bacteria, which were discovered to swim around cable bacteria as a nimbus, in enrichments with the freshwater cable bacteria *Candidatus* Electronema aureum GS (Thorup et al., 2021; Bjerg et al., in press). These flocking bacteria, or flockers, displayed chemotactic behavior toward the cable bacteria, without clearly touching or attaching to the filaments (Bjerg et al., in press). The flockers were only observed around actively electron-conducting cable bacteria, and when the cable bacteria were physically cut (removing the cable bacterium’s connection to oxygen and thus stopping electron conduction) the flockers dispersed (Bjerg et al., in press). The flocking bacteria are proposed to be aerobes, yet they were discovered in a hypoxic environment (suboxic/anoxic zone), where most high energy electron acceptors have been depleted (Bjerg et al., in press). The flockers are therefore hypothesized to donate electrons to the cable bacteria through interspecies electron transfer. Cable bacteria could be seen as an alternative electron acceptor (with a direct link to oxygen) for the flocking bacteria.

With this phenomenon’s very recent discovery in mind, any study into it is exploratory in nature. Little is currently known about what causes flocking and whether it is related to cable bacteria succession, for example, does it show up at a particular phase of the lifetime of a cable bacteria enrichment, or is it lifetime-unrelated? So far, flocking has only been observed when cable bacteria were very abundant and active, suggesting that the interaction is a consequence of some limitation of cable bacterial growth. To test this hypothesis, we set out to elucidate the occurrence of flocking and its relationship to cable bacteria succession, activity, and geochemical changes. We combined microsensor measurements in sediment cores with sequencing, to describe the biogeochemical succession of a single strain enrichment of *Candidatus* Electronema aureum GS (Thorup et al., 2021), including their activity (inferred from an electric potential (EP) microsensor), changes in pH profile, and relative abundance of cable bacteria. This was compared to microscopy observations of flocking behavior around cable bacteria from the profiled cores, in two replicate time series (TS1 and TS2) each lasting 3 months to resolve how often flocking appears and to determine the amount of flockers and their morphology.

## 2. Materials and methods

### 2.1. Sediment preparation, inoculation, and incubation

Black freshwater sediment was retrieved, in June 2019 (TS1) and October 2020 (TS2), from a pond at Aarhus University campus (Vennelyst Park), Denmark (56.164672, 10.207908) at a water depth of 0.5–1 m. The sediment was stored with overlying water at 15°C for 2 weeks.

Before inoculation, sediment was homogenized, sieved (pore size: 0.5 mm) and autoclaved in 2 L bottles for 20 min as described in Thorup et al. (2021) and Marzocchi et al. (2022). Cooled down sediment (15°C) was distributed into 20 and 10 ethanol-cleaned Plexiglas core liners that were closed with a rubber stopper at the bottom. After 24 h settling time, the stoppers were pushed upwards to align the sediment surface with the core liner edge. The cores were inoculated by transferring a clump of sediment from a two-week-old, pre-grown single-strain enrichment culture of *Ca.* Electronema aureum GS (Thorup et al., 2021) and submerged in an aquarium with autoclaved tap water. The aquarium was covered with aluminum foil to prevent algae formation, equipped with aeration and a lid to prevent excessive evaporation, and kept at 15°C. Overlying water was replenished and refreshed several times during the incubation periods.

During the first time series (TS1), O_2_, pH, and EP profiles were measured combined with 16S rRNA sequencing and microscopy observations over 80 days, due to technical issues, the videos could not be used for flocking observations. The sequencing and microscopy observations were repeated in the second time series (TS2) with O_2_ and EP measurements over 81 days, and additionally the pH was measured on day 81. Following microsensor measurements (of both TS1 and TS2), 1–2 of the measured cores were sliced based on geochemical zone: the oxic layer (determined by the oxygen penetration depth) and the anoxic layer (below the oxygen penetration depth). Samples for light microscopy and 16S rRNA amplicon sequencing were taken from the anoxic layer of each core, of which the latter were frozen at −80°C until RNA extraction.

### 2.2. Microprofiling and analysis

At each time point, profiles were made of 1–3 randomly chosen core(s). Microprofiles were measured, with the aeration turned off, by moving in-house made O_2_, pH and EP microsensors (Revsbech and Jørgensen, 1986; Revsbech, 1989; Damgaard et al., 2014) stepwise (100–500 μm) downwards from ~3,000 μm above the sediment–water interface (SWI) to a depth of ~35,000 μm using a motorized micromanipulator (Unisense A/S, Denmark). At each step there was a 2 s waiting time before measuring a value for 2 s. All profiles were acquired using a four-channel multimeter with built-in A/D converter (Unisense). For the EP and pH sensors an in-house made millivoltmeter (resistance >10^14^ Ω,), and for the O_2_ sensor a picoamperemeter (Unisense) that was digitized with a 16-bit A/D converter (ADC-216, Unisense), was used. The software SensorTrace PRO (Unisense) was used to control the micromanipulator and acquire measurements.

EP was used as a proxy for cable bacteria activity (Damgaard et al., 2014) and was measured downwards combined with an upwards profile. EP and pH profiles were measured against a general-purpose reference electrode (REF201 Radiometer Analytical, Denmark).

O_2_ profiles were calibrated using Na-ascorbate in alkaline anoxic tap water and air-saturated (15°C) overlying water. The pH sensor was calibrated by a 3-point calibration (pH 4.0, 7.0, 10.0) with AVS TITRINORM buffers (VWR Chemicals, Denmark), and pH profiles were always measured in the dark. The reach of the anoxic zones was derived by determining the oxygen penetration depth from the O_2_ measurements after the profiles were adjusted to the SWI. Two EP profiles (downwards and upwards) were made for each core, and were drift corrected, using the measurements from the overlying water and the time between the up and down profiles. Current densities were calculated per core and averaged per time point (1–3 profile pairs/cores), as showcased in Risgaard-Petersen et al. (2015), using a sediment conductivity of 0.04 S m^−1^ (water conductivity of 0.05 S m^−1^ and sediment porosity of 0.87).

The cathodic oxygen consumption (COC) of cable bacteria, which is the contribution of the cable bacterial cathodic cells to the total oxygen consumption performed in the sediment, and the (electric) current density (mA m^−2^) in the sediment generated by the cable bacteria, used for this purpose were calculated as described in Risgaard-Petersen et al. (2015) from the EP and O_2_ microsensor measurements of the exact cores that were subsequently sampled for flocking observations.

### 2.3. Cable bacteria rRNA sequencing

Samples from the anoxic zone, in duplicate when possible (at certain time points laboratory access time was limited due to COVID-19 prevention measures), underwent three freeze–thaw cycles increasing from 0.5 to 1.5 h. RNA was extracted using RNeasy Powersoil Total RNA kit (Qiagen), with DNase treatment (Turbo DNA-free kit, Ambion) and confirmation of RNA contents with Qubit (Thermo Fisher Scientific). Reverse transcription and PCR amplification of cDNA was performed with the OneStep RT-PCR kit according to instructions, RNase inhibitor or 5× Q-solution addition were not carried out (Qiagen), using primers Bac341F, Bac805R (Herlemann et al., 2011), 55°C annealing temperature and 30 PCR cycles. The 16S rRNA libraries were prepared according to the manual for 16S Metagenomic Sequencing Library Preparation (Illumina, United States), using AMPure XP Bead 0.8× and 1.12× (Beckman Coulter) for PCR clean-up. The 16S rRNA libraries were 2 × 300 bp paired-end sequenced on the Illumina MiSeq platform following the standard Illumina protocol for MiSeq reagent kit v3 (Illumina, United States).

Data processing started by removing low-read (<500) samples. Amplicon data was primer-trimmed with cutadapt (Martin, 2011). The DADA2 (Callahan et al., 2016) manual was followed with slight deviations; read dereplication after read clustering and read length filtering before chimera removal were added. Trimming was performed based on quality of the sequencing run and paired end merging was done with minimal overlap of 12 bp and 0 mismatch allowance. The Silva 138.1 database (Quast et al., 2012) was used for classification of the ASVs (amplicon sequence variants) to species level. Cable bacteria percentages of *Ca.* Electronema were extracted with Phyloseq (McMurdie and Holmes, 2013). 16S rRNA sequences from both time series experiments can be found under accession number PRJNA837365 in the Sequence Read Archive (SRA) at the NCBI.

Correspondence between the current density and relative abundance of cable bacterial 16S rRNA were determined by calculating the correlation coefficients for these two datasets with Pearson’s correlation method in R, on the datasets from TS1 and TS2.

### 2.4. Microscopy of flocking bacteria and video analysis

During incubation, the population of cable bacteria and the presence of flocking bacteria was followed using phase contrast microscopy. For this, we used in-house made ‘trench slides’ as described in Thorup et al. (2021). The central trench of the microscopy slide was filled with sediment from the anoxic zone, and the slide was covered with anoxic tap water and a coverslip. In the second time series, the edge of each trench slide coverslip was covered with polydimethylsiloxane (PDMS) as a seal against evaporation and to minimize water movement. The trench slides were kept in an enclosed chamber with a moist paper cloth to prevent dehydration at 15°C for the first day and subsequently at room temperature. These trench slides were then observed for up to 4 days for the presence of flocking bacteria. Microscopy was performed at room temperature using a ZEISS Observer Z1 (Zeiss, Göttingen, Germany) inverted microscope equipped with a phase contrast lens and a PALM automated stage. Images and videos were taken at 100, 200, or 400× magnification, with the Zen Black edition software (Zeiss, Germany) over 1–8 h.

To assess the presence of flocking bacteria, 5 trench slides were prepared per time point during TS2 and observed intermittently over a period of 4 days. Flocking behavior is hard to discern the first day after preparing a trench slide, because gradients of oxygen, sulfide need to establish, so that the cable bacteria can align themselves from sulfidic to oxic zone, and the two zones need to be delineated by a microaerophilic veil (Scilipoti et al., 2021; Bjerg et al., in press). Presence of flocking was therefore, with a few exceptions (days 2, 27, and 32), assessed on the second and fourth day of the trench slides incubation.

Bjerg et al. (in press), clearly describe that the flocking behavior of bacteria occurs around on the anoxic side of the microaerophilic veil of the free-laying cable bacteria spanning the oxic and anoxic zone on trench slides (Supplementary Figure S1). However, sometimes the presence of multiple cable bacteria hinders visual inspection of the flocking behaviors, although a haze of bacteria is often seen (Supplementary Figure S1). The presence of higher bacterial abundance (>100) around a cable bacteria can overcome this shortcoming. Based on the aforementioned previous observations, the following set of criteria was defined to confirm the presence of bacterial flocking behavior: (1) the presence of free-laying cable bacteria spanning from the oxic zone to the anoxic zone, and (2) high bacterial count around the cable bacteria (>100; Supplementary Figure S1). The detection of the phenomenon is therefore a conservative estimate of its prevalence, especially at time points where cable bacteria density on the slides was high. Each slide was inspected by following the microaerophilic veil clockwise around the slide. The microaerophilic veil was discerned by its high visibility as a dense collection of chemotactic bacteria that represent a quivering line of fast swimming bacteria that follow their preferred oxygen concentrations and the shape of the trench slide. A slide would only be marked as having flocking present if any cable bacteria fit both abovementioned criteria.

Videos for counting and morphology were recorded with frames 88 milliseconds apart. Only videos of sufficient quality, length, with a single cable bacterium present, and without major sediment particles, were used for subsequent analysis in FIJI (Schindelin et al., 2012). Videos were processed by removing the median pixel value from each pixel, in essence removing any parts of the video which were not moving. Morphology was assessed by eye, while length and width were measured using the line tool (FIJI). Counts were done by selecting a random 50 μm segment along the length of the cable bacteria filaments and counting all moving bacteria within 50 μm from the cable bacteria filament, i.e., all moving bacteria in a 100 × 50 μm box. For morphology counts, 5 categories were used: spirillum (short spirals with at least one full turn of the helix, and no more than 6), coccus (ovoids, almost as wide as long), spirochaete (at least 20 μm long, and more than 6 helical turns), rod (predominantly large, curved rods at least 4 μm long), and vibrioid (small curved rods with a tapered end), however these cells were mostly below 0.8 μm wide and 1.5 μm long, and the shape was often difficult to make out. As a result, the vibrioid category encompasses any very small bacteria (≤1 × 1.5 μm). Morphology counts were normalized against cable bacteria cells for each video and averaged per time point.

## 3. Results

### 3.1. Geochemical development in freshwater sediment

**Figure 1:**
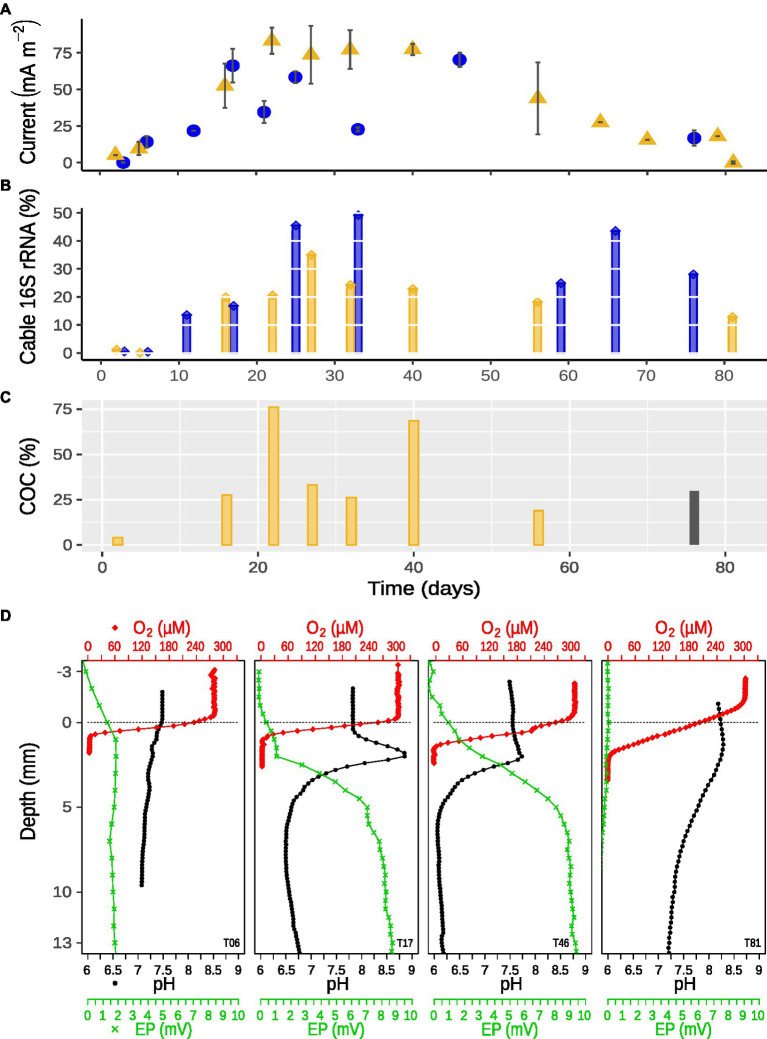
**(A)** Cable bacteria activity over 81 days of incubation displayed in current density (mean ± SD, *n* = 1–3 pairs of up and down microprofiles) between sediment surface and maximum EP within the sediment; blue circles, first time series (TS1); yellow triangles, second time series (TS2). **(B)** Relative abundance of cable bacteria 16S rRNA in anoxic zone over time; blue, first time series (TS1); yellow, second time series (TS2). **(C)** Cathodic oxygen consumption (COC) of cable bacteria over time calculated from the cores sampled for microscopy during TS2 (yellow); gray, calculated from TS1. **(D)** Examples of depth profiles of oxygen (red diamonds), pH (black dots), and EP (green crosses) at day 6, 17, 46 (TS1), and 81 (TS2). The dotted gray line indicates the sediment surface.

The two time series incubations (TS1, TS2) proved to be comparable in their geochemical developments (Supplementary Figure S2). Differences in the current density (activity), generated by the cable bacteria, between the two time series are visible. Yet, the in-and decreases in cable bacteria activity followed the same trend (Figure 1A; Supplementary Figure S2). The trend described low cable bacteria activity at the start (days 3–11), with no current on day 3 for TS1. From day 6 on, the activity increased to reach the highest current density (79 mA m^−2^) on day 22, then it appears to vary a little (days 26–40) before it slowly declines to zero (day 81; Figure 1A).

The cathodic consumption of oxygen (COC) by cable bacteria started low with ~5% (day 2) and increased to ~75.5% (day 17), followed by a dip toward 32%–26% and another increase to 65% (day 40), only to decrease to a trailing 14%–28% toward the end of the experiment (Figure 1C).

**Figure 2:**
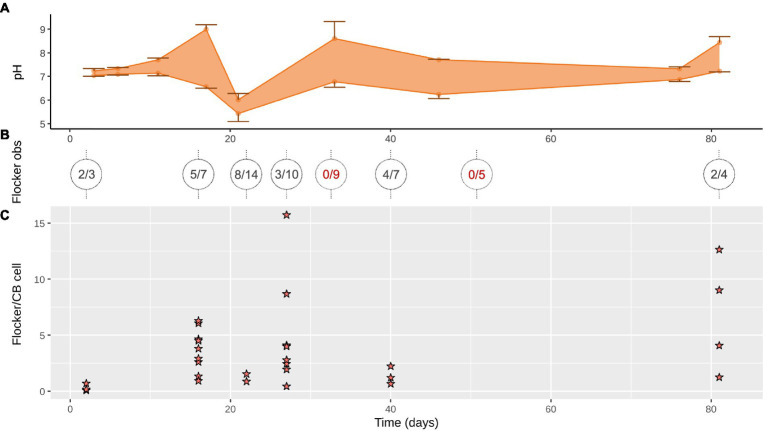
**(A)** Range of pH minima and maxima in the anoxic zone (down to 13 mm) where cable bacteria and flockers were observed (mean ± SD, *n* = 2–4). **(B)** Flocking observed over 1–4 days (on trench slides during TS2) in x/y where x is number of trench slides with successful flocking observations with y as total observation attempts, circles show individual time points with lines indicating the time points the sediment originated from; red, no detected flocking. **(C)** Flocking was often observed in multiple instances on a single trench slide, each instance is represented by a star. Each star indicates the number of flocking bacteria per cable bacterium cell within 50 μm on each side of the cable bacteria filament in a single still frame of a high-quality video of the given time point.

The OPD varied during the 81 days from ~1.5 ± 0.2 mm at the start, where after it decreased to ~0.5 ± 0.15 mm on day 33 (data not shown), only to deepen again at the end to ~3 mm (Figure 1D). The depth of the cable bacteria activity based on the EP, increased from 1 mm (day 6) to 6 mm (day 17), and further increased to a maximum depth of 10–13 mm (day 33; data not shown), before retreating to 7 mm (day 46), ending in non-detectable EP (day 81; Figure 1D). The cable bacteria-created pH peak and minimum were not visible until day 11 and were strongest between day 17 and 33 (9 and 5.5), while the acidity mellowed out at the end, the pH peak increased again to around 8 (Figures 1D, 2A).

### 3.2. Cable bacteria relative abundance

A trend similar to that of the activity and geochemistry development was present in the relative abundance of the 16S rRNA specifically for *Ca.* Electronema aureum GS (Figure 1B). Cable bacteria relative abundance started with a near-zero lag phase (day 2–6) followed by a strong incline until they reached 49 and 37% (TS1, TS2) of the sequenced 16S rRNA as the highest relative abundances (TS1: day 33, TS2: day 27). The relative abundance of cable bacteria declined very slowly to 13% (day 81), but never reached zero within this timeframe (Figure 1B). At every sampled time point, cable bacteria rRNA was present (Figure 1B). This corresponded with microscopy observations of moving, living, cable bacteria filaments at each time point in both time series experiments (Supplementary Figure S3). Clear differences between amounts of actively moving (and therefore presumed to be living) cable bacteria were seen during microscopy observations (Supplementary Figure S3).

### 3.3. Flocker occurrence and morphology

**Figure 3:**
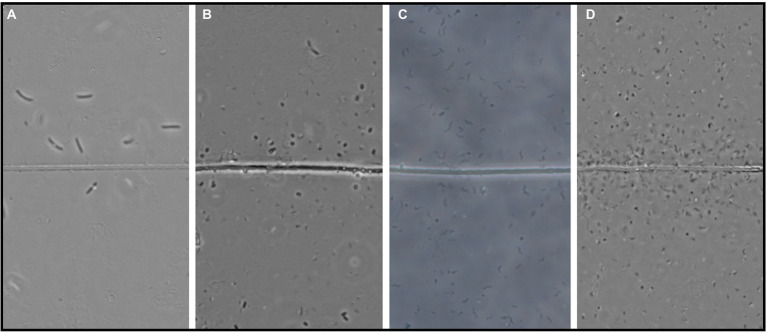
Four examples of the observed flocker morphologies. **(A)** Large curved rods, **(B)** Coccoids, **(C)** Spirillae, and **(D)** Vibrioids. “Line” in the middle is the cable bacteria filament. Scale bar, 5 μm.

**Figure 4:**
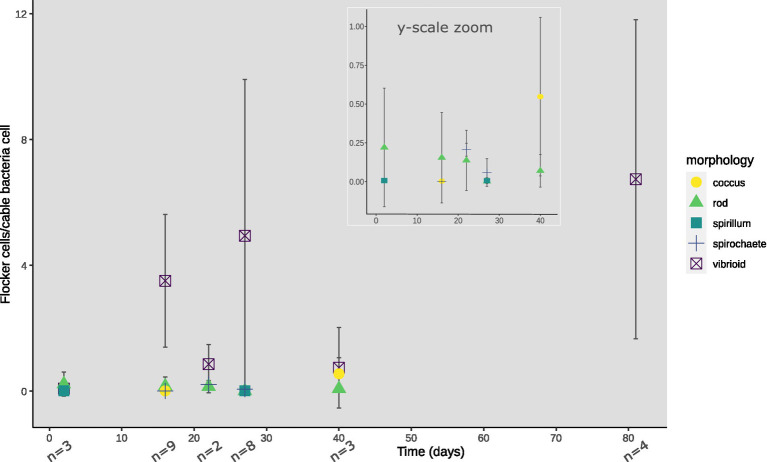
Morphologies of flocking cells normalized against cable bacteria cells observed per time point of TS2. Inset shows y-axis zoomed onto 0–1.0 flocking cells/cable bacteria cells until day 40 for low abundancy flocking morphologies. Absence of a morphology-indicator at a certain timepoint depicts zero observed cells of that morphology, with n showing the number of video-frames used for cell counting.

At every time point, except for day 33 and 56, flocking was observed during one or more of the 4 observation days, and most often on days 17, 22 and 40 (Figure 2B). The number of flocking cells per cable bacteria cell varied between observations of individual flocking occurrences, but also between time points from ~1–2.2 at the lowest (days 2 and 22), to ~6.8 (day 12), with the highest counts observed as ~12.6 and ~16 (day 81 and 27; Figure 2C). Besides occurrence and number of cells, we derived cell shape, width, and length from the microscopy observations. Five different cell morphologies were identified: rods, cocci, vibrioids, spirochaetes, and spirillae (Figure 3). Vibrioids appeared at every time point that had flocking and were the most abundant flocker morphology (0.08–6.7 cells/cable cell) at all observations except for day 2, when rods were most prominent (Figure 4). Rod shaped flockers were the second most abundant type (0.22–0.07 cells/cable cell) and were observed at every time point except for day 81 (Figure 4). Rod-shaped flockers also appear to decrease in amount over time from being the most observed morphology on day 2 to day 81, where the rod-shaped flockers were absent (Figure 4). Cocci only appeared on days 16 and 40. On the second day, 0.55 coccus-shaped flockers were observed per cable cell (Figure 4). The other morphologies were very rare or restricted to few time points (Figure 4). Cell sizes of the flocking bacteria ranged from tiny (~0.5 × 1.6 μm) to large cells (~1.4 × 6.1 μm; Supplementary Figure S4).

## 4. Discussion

### 4.1. Freshwater cable bacteria development

The geochemical development in our *Ca.* Electronema aureum GS enrichment is generally comparable to what was previously seen in cable bacteria succession experiments: a decrease in OPD, a pH peak at the near-surface and a pH minimum in the anoxic zone indicative of cable bacteria activity (Figures 1D, 2A,B; Schauer et al., 2014; Xu et al., 2021). This activity corresponded to the changes in cable bacteria density (Figure 1B; Supplementary Figure S2). The formation of a pH peak indicated rapid sulfide oxidation, resulting in more electrons to transport evident in the concurrent increase in current density, which eventually pushes the OPD toward the surface (Supplementary Figures 1B,D). The succession pattern of *Ca.* Electronema aureum GS rRNA showed an increase, then a long stable phase, followed by a decline, though this decline is slower than seen in other studies (Schauer et al., 2014; Xu et al., 2021). At every sampling point, cable bacteria were observed both in relative abundance numbers and to be moving in trench slides (Figure 1B; Supplementary Figure S3), suggesting that even in absence of a clear geochemical fingerprint, cable bacteria persist.

The cable bacteria’s COC shows that the majority of oxygen is consumed by cable bacterial respiration at these time points. This COC, combined with a retreating OPD, may push (micro-) aerobic bacteria, that live at the oxic-anoxic interface, to resort to alternatives such as donating their electrons through interspecies electron transport to the cable bacteria, instead of directly to oxygen. If donor concentrations are higher below the oxic-anoxic interface, this might be a favorable option for a flocking bacterium. Bjerg et al. (in press) suggested that the electron donors might be either sulfide, organic compounds or even Fe^+2^ with an unknown soluble mediator as an intermediary. A closer look at the gradients of these electron donors below the oxic-anoxic interphase might shed light on why these flocking bacteria are attracted to cable bacteria rather than trying to find oxygen.

### 4.2. Flocker occurrence and morphology

In each observation of flocking, we identified different morphologies and sizes of flockers as previously described by Bjerg et al. (in press). Out of the five different morphologies of flockers, the most prominently present were vibrioids, however this category also encompassed cells that were too small to determine differences in morphology by light microscopy (Figures 3, 4). The second largest group was described by rod-shaped flockers and followed a downward trend while the vibrioids were increasing over time and were the sole morphology observed on day 81 (Figures 3, 4). The other morphologies: cocci, spirilla, and spirochaetes, were only sporadically present and in low abundances (Figures 3, 4). The different morphologies we described were present at almost every time point in this study, which shows that the flocking community was not made up of a singular species of bacterium (Figure 4). Most of the observed flocking cells were small (0.5–1 × 0.9–2 μm; Supplementary Figure S4). Cell sizes of flockers that we recorded in this study diverge from Bjerg et al. (in press), who reported more medium sized cells (1 × 2–3 μm) than we do here (Supplementary Figure S4). Such a clear majority of small cells could be explained by a lower motility cost for smaller cells (Mitchell, 2002). The diverse cell sizes and morphologies do not provide specific taxonomic information but underline previous findings of high taxonomic diversity among the flockers, which suggests that they use a common mechanism of interspecies interaction. One such mechanism could be interspecies electron transfer, provided by electron shuttles not produced by specific bacterial species but intrinsic to the sediment, for example humic substances or flavins (Monteverde et al., 2018; Bjerg et al., in press).

Contrary to our initial expectation, flocking was present for the majority of the cable bacteria’s succession in the sediment (Figures 1A,B, 2C). Flocking appeared already on day 3 of the cores’ lifetime suggesting that the flocking bacteria are carried over when the core is inoculated. Cable bacteria are unlikely to be limited in electron donors at such an early stage in the life of the core. This is evident from the rapid increase in cable bacterial density in the first days and suggests an exponential increase congruent with low to no resource limitation. Flocking thus does not appear limited to periods of low electron donor availability for the cable bacteria. The disappearance of flocking on days 33 and 56 is puzzling, as these appear not any different from days where flocking is present, according to the measured parameters. This absence could be due to the limitations of flocking detection, as the trench slides became very crowded around 20–40 days into the incubation period (Supplementary Figures S1, S3), and very seldom free-laying cable bacteria were encountered. Finally, flocking was observed on the last days of the incubation. Non-moving cable bacteria, without connection to oxygen, and sometimes fragmented with partially degraded cells (therefore presumed dead), were often encountered in these trench slides, likely washed out from the central sediment trench when the slide was made. Flocking was never observed around these dead cable bacteria but was very prevalent around the few remaining living filaments, which were characterized by their high motility, length, and with almost always flocking on them. If the flockers were beneficial to cable bacteria, this could explain the perceived health of these surviving filaments, suggestive of the flockers’ providing electron donors to the cable filament at what is likely starving conditions. Alternatively, a parasitic relationship between flocker and cable bacterium would have the flockers attracted to the few remaining active cable bacteria in the slide.

There is no apparent pattern between the measured parameters in this study: pH, EP, O_2_, and the appearance of flockers around cable bacteria. This strongly suggests that flocking is governed by parameters not measured yet or not measured at sufficiently fine scale. Flocking occurs in a zone around 50–100 μm distance from a cable bacterium (Bjerg et al., in press). It is currently unknown and difficult to measure how the fine scale gradients in the trench slides directly around the cable bacteria filaments and thus in the ‘flocking ranges’ are. Especially, when we consider the cable bacterial metabolism of sulfide oxidation, strongly influencing both sulfide and sulfate availability and pH due to proton release within the “flocker range,” possibly generating strong gradients close to the filaments. A better understanding of the fine scale gradients of pH and sulfide around the cable bacteria, in the flocking area, which are created by the cable bacteria’s metabolism would tell us whether these environmental parameters are relevant for the flocker community. It may be more useful to do manipulation experiments in the starvation phase near the end of the incubation as a response to addition of a suitable electron donor for cables, or flocking bacteria should be more evident. With flocking showing up so early and so late in the incubation’s timeline, it is increasingly likely that any electron shuttle is endemic to the sediment, rather than produced by cables or flockers. Electron shuttle usage is highly dependent on the presence of shuttles, therefore we suggest to measure this as a parameter, combined with on-slide fine scale geochemistry, to get a step closer to whether flocking is a form of shuttle-based IET.

### 4.3. Conclusion

*Candidatus* Electronema aureum GS showed activity and succession patterns comparable to enrichments of marine and freshwater cable bacteria populations.

Diverse flocking bacteria were observed around cable bacteria throughout the succession of *Ca.* Electronema aureum GS, independent of relative abundance or sediment pH. Our main conclusion is that as long as there are actively electron-conducting cable bacteria filaments present, flockers can be seen around these.

## Data availability statement

The datasets presented in this study can be found in online repositories. The names of the repository/repositories and accession number(s) can be found in the article/Supplementary material.

## Author contributions

JL and LB conceived experiments. JL, LB, JB, and AS designed experiments. JL, LB, and JB conducted experiments. JL, JB, LB, IM, and LN performed the analyses. JL, JB, LN, AS, and IM wrote the manuscript with input from all authors. All authors contributed to the article and approved the submitted version.

## Funding

This research was financially supported by the Danish National Research Foundation (grant DNRF136 to LN) and the Carlsberg Foundation (grants CF19-0666 and CF21-0409 to JB).

## Conflict of interest

The authors declare that the research was conducted in the absence of any commercial or financial relationships that could be construed as a potential conflict of interest.

## Publisher’s note

All claims expressed in this article are solely those of the authors and do not necessarily represent those of their affiliated organizations, or those of the publisher, the editors and the reviewers. Any product that may be evaluated in this article, or claim that may be made by its manufacturer, is not guaranteed or endorsed by the publisher.
