# Supplementary material for: Persistent flocks of diverse motile bacteria in long-term incubations of electron-conducting cable bacteria, Candidatus Electronema aureum
Source: Front Microbiol. 2023 Feb 23;14:1008293. doi: 10.3389/fmicb.2023.1008293 (PMC9998039; doi:10.3389/fmicb.2023.1008293)
Supplement: Supplementary file 1 [file Data_Sheet_1.PDF]

Supplementary Material

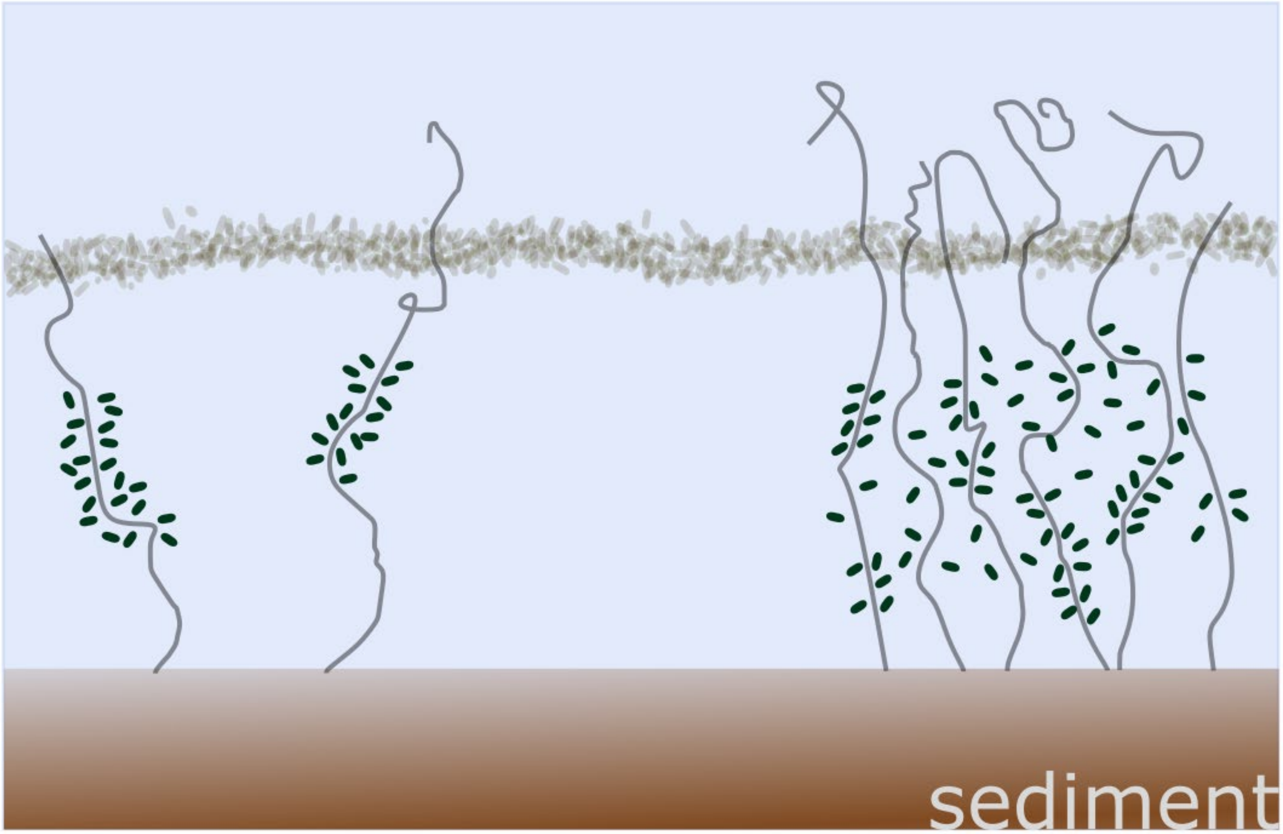

Supplementary Figure 1 - Theoretical overview of our definition of flocking with regards to overcrowding of cable bacteria by a possible flocking event. Left side, cable bacteria lay at sufficient distance to determine individual flocking appearances. Right side, slide too crowded with cable bacteria to determine presence or absence of flocking.

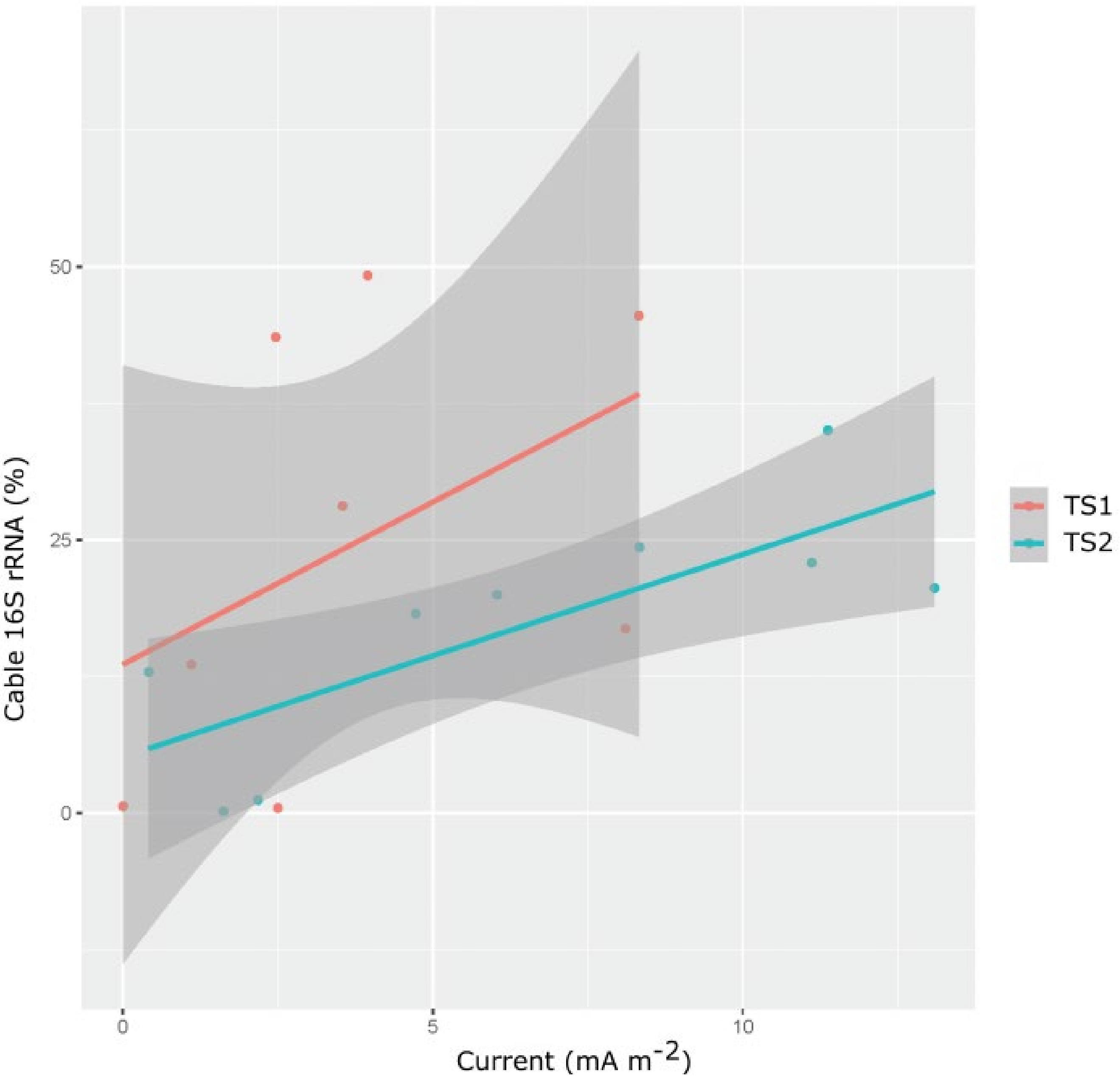

Supplementary Figure 2 - Pearson's test for correlation of coefficients between Cable 16S rRNA (%), relative cable bacterial abundance, and Current (mA m^{-2}), current density, as the data was found in Figure 1 of this manuscript. TS1 experiment in red (with insufficient data rho = 0.454 and p = 0.258) and experiment TS2 in blue (with rho = 0.78 and p = 0.013), showing a strong positive correlation.

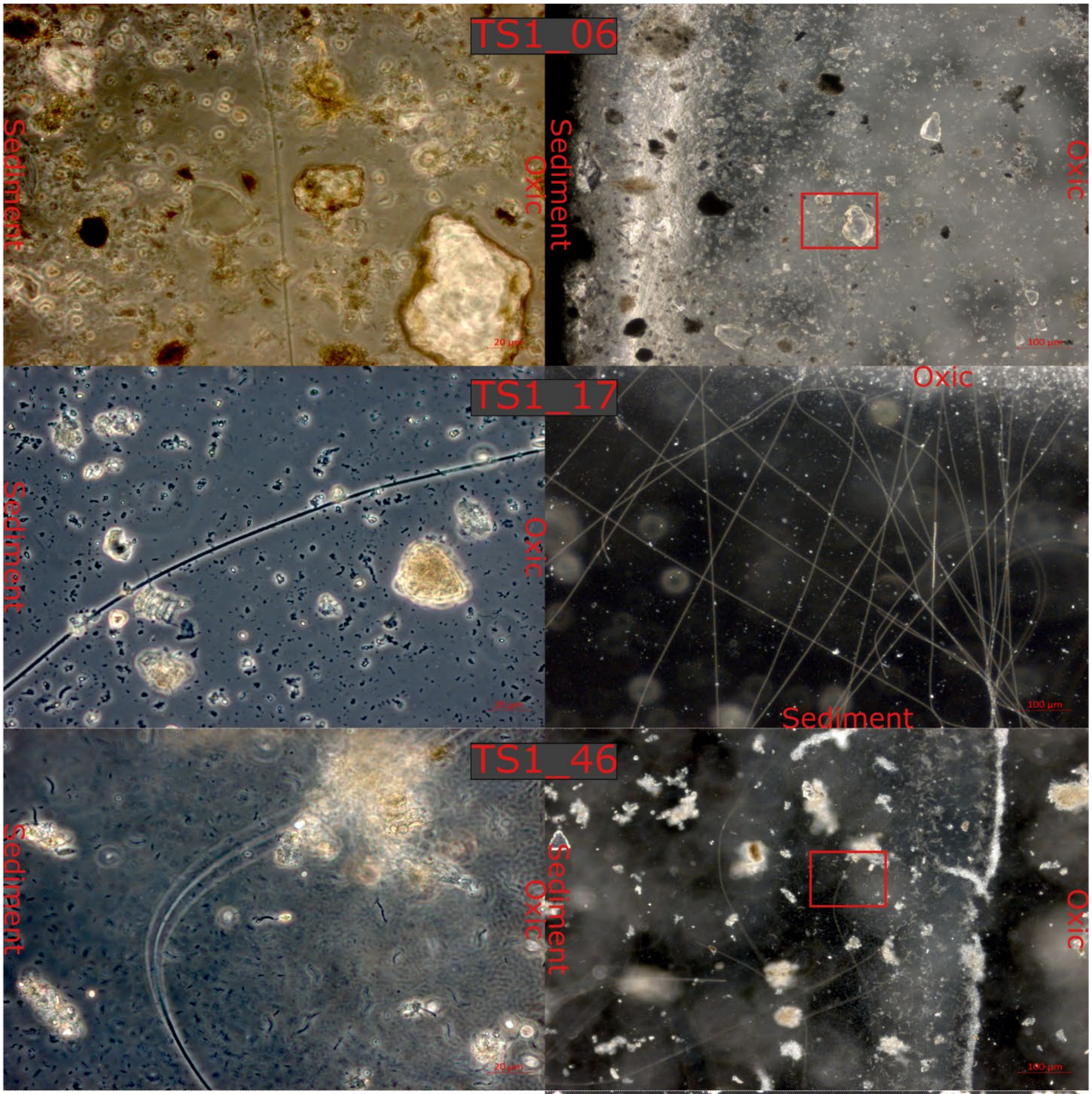

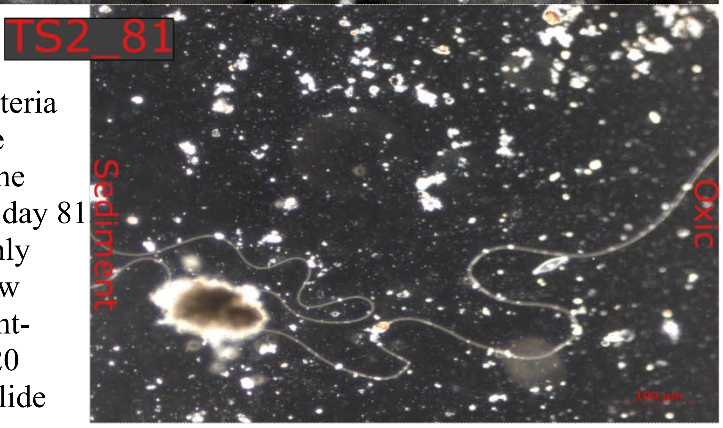

Supplementary Figure 3 - Examples of cable bacteria at day 6, 17, 46, and 81. Showing that on day 6, almost no cable bacteria were visible. On day 17 and 46, many cable bacteria that span from sediment to oxic zone were present and actively moving. And, on day 81 very few cable bacteria were present and only slowly moving. Left-hand micrographs show health of individual cables, zoom-ins of right-hand marked by red rectangles (scale bars 20 µm), right-hand micrographs show trench slide overviews (scale bars 100 µm).

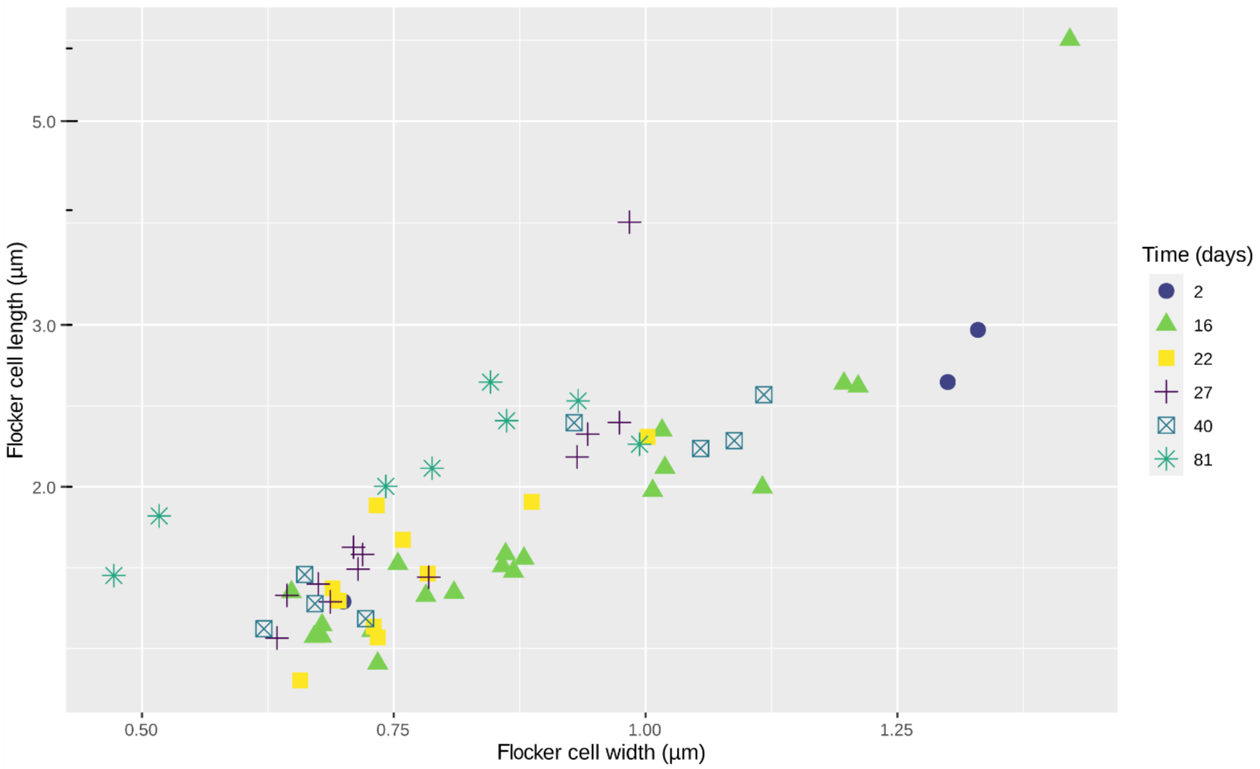

Supplementary Figure 4 - Unique sizes of the flocking cells by cell length and width. Only the first occurrence of each distinct cell size was recorded per time point. Y-axis is logarithmic.
